# Raman temperature and density measurements in supersonic jets

**DOI:** 10.1007/s00348-021-03162-2

**Published:** 2021-03-06

**Authors:** Mark P. Wernet, Nicholas J. Georgiadis, Randy J. Locke

**Affiliations:** 1 NASA Glenn Research Center, Cleveland, OH 44135 USA; 2 HX5, LLC, Cleveland, OH 44135 USA

## Abstract

Prediction of flow-field properties in supersonic jets using computational fluid dynamics (CFD) code predictions has become routine; however, obtaining accurate solutions becomes more challenging when there is a significant temperature difference between the jet core and the ambient air and/or compressibility effects are significant. Benchmark sets of flow field property data are required in order to assess current CFD capabilities and develop better modeling approaches for these turbulent flow fields where accurate calculation of temperatures and turbulent heat flux is important. Particle Image Velocimetry, spontaneous rotational Raman scattering spectroscopy, and Background-Oriented Schlieren (BOS) have been previously used to acquire measurements of the mean and root-mean-square (rms) velocities, the mean and rms gas temperatures, and density gradients in subsonic jet flows and film cooling flows. In this work, the ability to measure density is added to the list of measurands available using the acquired Raman spectra. The suite of measurement techniques are now applied to supersonic jet flows. The computation of the local gas pressure in the potential core of an over-expanded jet is demonstrated using the Raman measured gas temperature and density. Additionally, a unique density feature in temperature matched, perfectly expanded jet flow shear layers identified using BOS was verified using the Raman measurement technique. These non-intrusive flow measurements are compared against RANS predictions of the supersonic jet flow properties as a means of assessing their prediction accuracy.

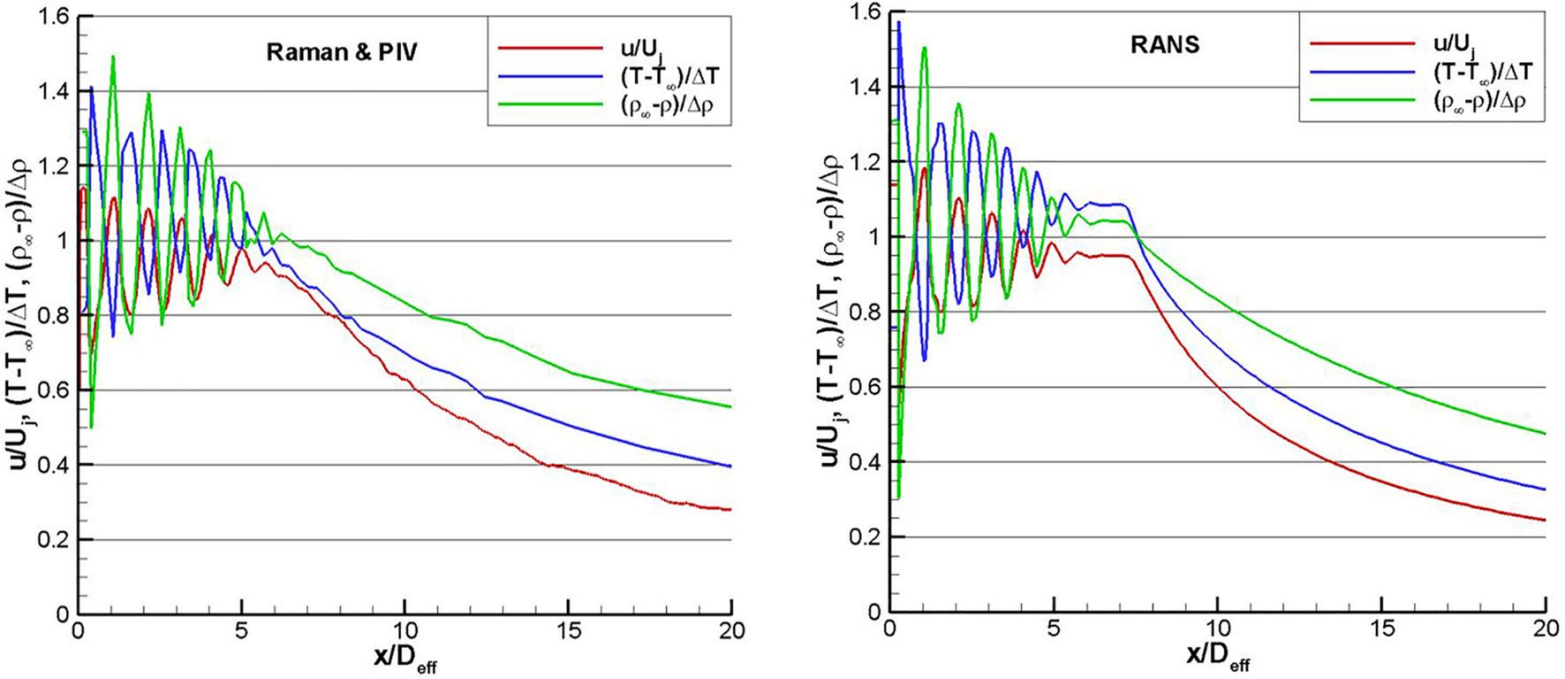

## Introduction

Subsonic and supersonic jets have been extensively investigated using probes (temperature and pressure) and hot wires (Lawrence 1956; Bradshaw et al. 1964). While probes can be operated over a wide range of flow conditions, hot wires are typically limited to flows below Mach 0.5. Extracting velocity data requires two different probe measurements (total pressure and total temperature) which are not acquired simultaneously. Further, extraction of accurate velocity data in supersonic flows requires the determination of a recovery factor to complete the data reduction (Albertson et al. 1993; Staff 1953). Non-intrusive optical diagnostics avoid these complications in addition to not disturbing the flow field under study. In a pioneering research effort, probes, hot wires, and the then newly developed Laser Doppler Velocimetry (LDV) technique were used to characterize subsonic and supersonic nozzle flows with both hot and cold gas exit conditions (Lau et al. 1979; Lau 1981). These data were used to develop correlations of potential core length and width with respect to Mach number using the measured nozzle flows from *M* = 0.5 up to *M* = 1.67. Lau’s work established the benefits of optical diagnostics over probes, especially in supersonic flows. Panda and Seasholtz (1999) used spectrally resolved Rayleigh scattering to make point measurements of velocity and temperature in several jets, including supersonic jet flows. The work of Seiner et al. (1992) yielded unique velocity and temperature measurements in a Mach 2 nozzle. Even though they were acquired using probes, these data still remain some of the highest jet exit temperature data available in the literature.

The non-intrusive point-based measurement techniques were soon eclipsed by planar techniques. Bridges and Wernet (2003) compiled an extensive set of velocity data using Particle Imaging Velocimetry to characterize the SMC000 (Simple Metal Chevron) nozzle at a series of subsonic jet Mach numbers and operating temperatures. These data have been used extensively by the jet aerodynamics and aeroacoustics CFD communities. More recently, the SMC000 nozzle was investigated using Raman spectroscopy to collect the mean and rms temperatures (Locke et al. 2017) in order to augment the existing database of PIV velocity measurements.

PIV is the de facto standard for measuring flow velocities and is readily applied in the high-temperature, supersonic flows encountered in this work (Bridges and Wernet 2003). The Consensus data set of PIV, LDV and probe measurements is widely used for CFD development (Bridges and Wernet 2011). While PIV systems are ubiquitous and commercially available, there are limited options for acquiring non-intrusive temperature and density measurements. Panda and Seasholtz (2000, 2005) used spectrally resolved Rayleigh scattering to make point measurements of velocity, temperature, and density in medium scale supersonic jet flows with very controlled conditions and a co-flow of filtered air to eliminate particulates. Rayleigh spectroscopy was also used in large-scale jet flow facilities to measure the flow parameters such as velocity, density and temperature (Mielke and Elam 2009). While the velocity and density data were of good quality, the rms temperature data are of lower quality due to contamination of the measurements by entrainment of particulates in the flow. The Rayleigh technique also requires a complex optical system setup and is very sensitive to ambient light, flare light, and vibration. Coherent Anti-Stokes Raman Spectroscopy (CARS), a complex optical technique requiring precise alignment of three coincident laser beams, has been used to obtain average temperature in high speed and reacting flows. The CARS technique is plagued by very complex optical setup and the associated alignment issues thereby requiring the laser to be remotely located in a climate-controlled room (Cutler et al. 2014; Tedder et al. 2009). The Transient Grating Spectroscopy (TGS) technique has been used to measure temperatures in jet flows (Kuehner 2010); however, it too suffers from a complex optical setup requiring precise alignment of multiple laser beams and as such is not amenable to large scale, outdoor facilities. Vibrational and rotational Raman spectroscopy are inelastic scattering techniques that have been used quite successfully to perform temperature measurements on a variety of flows. Single-shot vibrational Raman thermometry has been performed in flames, where a fast-optical shutter was used to minimize flame emission that would otherwise obscure or interfere with the SRS spectrum (Ajrouche et al. 2014). Vibrational Raman is ineffective in low temperature regimes due to the lack of the anti-Stokes bands; however, pure rotational Raman spectroscopy, which is not dependent upon anti-Stokes lines, has been used to interrogate expanding supersonic flows of CO_2_ for measuring temperature (Maté et al. 1998). Rotationally resolved Raman has more recently been used to acquire mean and rms temperature measurements in high-temperature flows, from subsonic to supersonic speeds in harsh real-world test facilities (Locke et al. 2017; Wernet et al. 2018, 2019, 2020). Rotationally resolved Raman scattering is the only technique which is relatively simple to setup/align and robust against the hostile environments found in real-world aerospace simulation facilities; therefore, it was the technique of choice for measuring the gas temperatures in supersonic jet flows of interest. The Raman temperature measurement diagnostic is extended in this work to also extract density data from the acquired Raman spectra. The ability to simultaneously measure temperature and density from the Raman spectra, which enables computation of the local static pressure, greatly increases the value of the measurements in cases where the pressure field must traditionally be assumed to be globally constant (Reid et al. 2011; Rekhy et al. 2020). In both the pressure-matched and especially over-expanded supersonic jet flows studied in this work, the pressure field inside of the potential core is not constant. Outside of the potential core and across the shear layer, the pressure is in equilibrium with the ambient and simultaneous measurements of temperature and density are redundant. However, inside the potential core, where the pressure may vary significantly, the simultaneous measurement of temperature and density enables computation of the local gas pressure, which to the best of the author’s knowledge, has not been previously reported in the literature.

The objective of this work is to apply multiple non-intrusive diagnostics (BOS, PIV, Raman temperature and density measurements) to characterize a pair of convergent-divergent supersonic nozzles designed for Mach 1.36 and Mach 1.63 at perfectly expanded conditions. The non-intrusive diagnostics provide both mean and rms measurements of the flow properties. PIV provides planar flow field measurements, yielding high spatial resolution measurements of a large region of the flow field. The Raman point-based diagnostic was used to acquire centerline, lip-line, and radial profiles thereby extending spatial range of flow field temperature and density properties beyond just the jet centerline. The matrix of flow conditions for this test was constructed to systematically investigate the effects of temperature and Mach number on the flow fields. A database of measurements, where only a single parameter is varied provides an invaluable resource for CFD prediction capability assessment and accurate turbulence model development. The full set of measurements from this work including a Mach 2.0 nozzle are available via Wernet et al. (2020).

## AAPL laboratory, SHJAR and experimental setup

All the data presented herein were obtained on the Small Hot Jet Acoustic Rig (SHJAR) located within the AeroAcoustic Propulsion Laboratory (AAPL) at NASA GRC. The AAPL is a 19.8 m radius geodesic dome with its interior walls covered by sound absorbing wedges providing a near anechoic environment. When in operation, the AAPL is open to the outside environment via the large, 17 m wide by 11 m high exhaust-door. The SHJAR is a single flow stream free jet rig capable of operating up to Mach 2 at jet static temperature ratios up to approximately 2.8. The centerline of the nozzle exit of the SHJAR is 3 m above the floor. Vitiated flow heated up to 950 K is provided by an inline hydrogen combustor and supply air is provided by central compressor facilities, permitting continuous operation. The fuel–air mass-flow ratios for heating the supply air ranged from 1.3 × 10^–3^ to 8.2 × 10^–3^ resulting in a 10–20% reduction in the mass of oxygen in the vitiated air at the nozzle exit, but not large enough to cause any significant difficulties in processing the acquired Rotational Raman spectra and extracting gas temperature estimates.

The initial nozzle designated as SMC000 used in the SHJAR was a convergent only reference nozzle, (Bridges and Wernet 2003). In later work at NASA GRC, supersonic nozzles up to a perfectly expanded Mach number of 1.8 were evaluated using PIV (Bridges and Wernet 2008). Both of the convergent-divergent nozzles used in this study have an exit diameter of 50.8 mm and an overall length of approximately 165 mm. The external dimensions of the nozzles are identical, only the internal contours are different in order to meet their perfectly expanded condition. The SMC015 nozzle has a perfectly expanded Mach number of 1.36, SMC017 has a perfectly expanded Mach number of 1.63.

## Pure rotational raman temperature measurements

Rotationally resolved Raman scattering has been previously used to measure gas temperature in heated high-speed jet flows in the SHJAR facility at NASA GRC (Locke et al. 2017). A thorough discussion of Raman spectroscopy theory and practices can be found in Ferraro and Nakamoto (1994). In general, Raman scattering is an inelastic process, with a signal intensity approximately 10^–3^ times that from Rayleigh-scattered light. Raman scattering is not dependent on wavelength but is linear with respect to the species number density and is species specific by virtue of the quantization of individual molecular energy states. In this work, the composition of the gas is known (heated air) and only the gas temperature and density is being measured, which greatly simplifies the data analysis and reduction.

The key components of the Raman temperature diagnostic are the long pulse length laser and the collection optics. A 10-Hz Continuum long pulse length Agilite Nd/YAG laser with 600 mJ of energy at 532 nm spread across 200 nsec was used to probe the flow temperature and density while still avoiding breakdown of the gas. The 9-mm-diameter output beam was focused by a 500-mm spherical lens to a roughly 70 µm beam waist. In order to maximize the collected rotational Raman scattered signal, a pair of 135 mm Nikon collection lenses set at f/4 and equipped with 42-mm extension tubes were used to collect the scattered light. The lenses were vertically mounted and focused on the laser focal volume approximately 257 mm distant. Each lens was displaced from the horizontal plane and tilted (upper −8.5˚, lower + 8.5˚), which is the closest the lenses could be mounted side by side.

The light captured by each lens passed through a 532-nm RazorEdge long-pass 24.5-mm filter attached to the rear of each camera lens mount. The filtered light was focused onto a bifurcated fiber bundle from Fiberoptic Systems, Inc. The input ends of the bifurcated fiber bundle each contained fifty-seven 100-µm diameter cladding-free fibers, which were formed into a linear array at the output end of the bundle. The linear fiber bundle output was coupled to the entrance slit of an Acton 500-mm imaging spectrometer with an 1800 groove ruled grating and a wavelength centerline of 537 nm resulting in a spectral wavelength span of approximately 12 nm. The slit of the spectrometer was opened to its maximum aperture of 2.0 mm allowing the fibers to act as their own 100 µm slit. A PI-MAX2 ICCD camera with an 18-mm-diameter gated intensifier from Princeton Instruments was coupled to the exit of the spectrometer and the resultant rotational Raman spectra captured using Princeton’s WinSpec32 software. The Raman measurement volume is defined by the intersection of the laser beam diameter and the two 135 mm lens collection cones. The length of the 70-µm-diameter beam collected by the 135 mm lenses is defined by the size of the fiber bundle used to collect and transmit the light to the spectrometer. The entrance face of each of the fiber bundles is 0.85 mm in diameter. The 135 mm lenses image a 1.2 mm length of the laser beam onto the face of the fiber bundle. The resulting cylindrical probe volume used in this work is 4.5 × 10^–3^ mm^3^.

### Raman data processing: temperature

Estimates of the gas temperature are extracted from the acquired rotational Raman spectra using an iterative process, which determines the best fit between the measured spectrum and the spectrum computed using a pure-rotational Raman scattering model, which is described in more detail in Locke et al. (2017). The Raman scattering spectrum model assumes that the gas composition is a mixture of only molecular nitrogen and oxygen, which is justified since the measurements are made in air and other atmospheric gaseous components do not contribute significantly to the Raman signal. For a given temperature, the model computes Raman line locations and strengths in the Raman Stokes (S) bands of nitrogen and oxygen up to rotational quantum numbers of 50. The merged array of Raman lines for N_2_ and O_2_ with their respective strengths and wavenumbers, is then convolved with a Voigt kernel derived from the spectral profile of the pump laser wavelength peak as measured through the optical system.

At each measurement position, 1000 single-shot Raman spectra are acquired. Typically, there are several bad spectra due to the wait time of the camera/spectrometer/image intensifier. Additionally, there are times when particulates in the flow passed through the measurement volume producing strong signals at the laser line wavelength, yielding Rayleigh/laser line peaks of higher intensity than the Raman signal. In order to remove these spurious spectra, the data set is sorted to remove the 10 highest amplitude spectra. The number of particulate contaminated spectra removed was adjusted according to the number of particles present during the data acquisition. Hence, a maximum of 990 single-shot spectra were used in each ensemble.

**Fig. 1:**
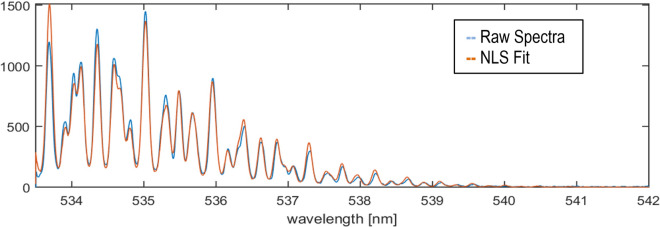
Sample averaged rotational Raman temperature spectrum with fit for a gas temperature of 547 K

The 990 spectra were then used to compute an average spectrum, which was fit to the O_2_/N_2_ model function using a MATLAB combined genetic algorithm for the global search followed by a local search using a nonlinear least squares (NLLS) routine. The model parameters used in the fit included the temperature, the Raman signal amplitude, the kernel width (a 2-parameter Voigt instrument function) and the spectrometer grating calibration parameters, which included the laser line center location. The fit of the mean spectra provided the initial estimates for all of the single-shot spectral fits. The estimated temperatures from the processed spectra were then used to compute the mean temperature estimate and the rms temperature across the ensemble, T′. The time to process the 990 spectra was on the order of 1 min on a 20-core CPU. A sample averaged spectrum acquired at a gas temperature of 547 K and its corresponding fit are shown in Fig. 1.

### Raman data processing: density

The acquired Raman spectra also contain information about the density of the gas in the probe volume at the time of the measurement. The collected signal energy is proportional to both the laser power and the number of scattering molecules in the probe volume at the time of the measurement. Normalizing the measured Raman signal level or the area under the Raman spectra by the energy in each laser pulse yields a measurement proportional to the gas density. A photodiode was placed near the laser exit aperture to collect light from each laser pulse. The photodiode signal was digitized using a PicoScope high-speed digitizer, which was synchronized to the laser pulse output using the PI-Max2 camera intensifier gate pulse as a trigger signal.

### Raman system temperature calibration

**Fig. 2:**
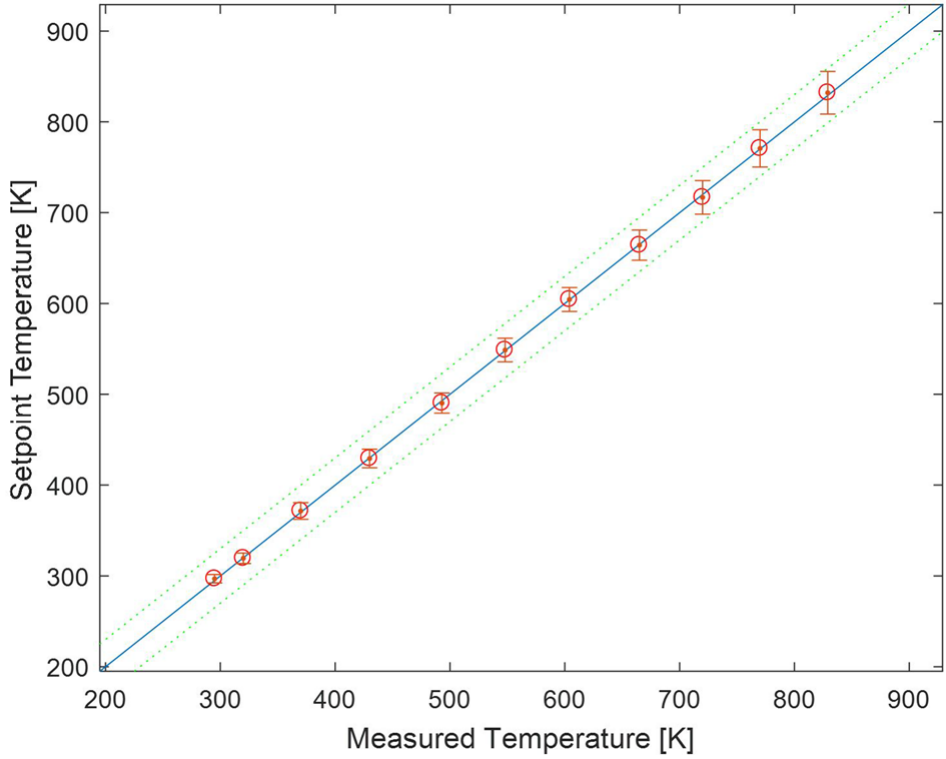
Plot comparing the thermocouple reading versus the calculated temperature and the RMS variations in temperature (plotted as error bars) over the range of calibration temperatures

The Raman technique samples the molecules in the probe volume in order to estimate the gas temperature. The molecules in the probe volume can have any one of a number of states as defined by the Boltzmann distribution. The number of populated states increases with temperature, and hence the rms temperature increases with increasing temperature of the gas. This is a known and documented characteristic of the technique, which is actually a systematic measurement error (Locke et al. 2017). A calibration of the Raman system in a well characterized environment free from flow turbulence of other noise sources is required in order to characterize this inherent rms temperature variation in the technique. A lab scale setup using the concentrated output from an electrical heat gun calibrated using a thermocouple was used to acquire the calibration data (Locke et al. 2017). The Raman spectra acquired at 11 different temperature settings on the heat gun were processed to extract the mean and rms gas temperature. Figure 2 shows the mean temperature of all 990 accumulated spectra along with the rms error bars at each temperature. The mean calibration temperature measurements are found to be accurate to < 0.2% over the range of 296–850 K. Here accuracy is defined as the deviation of the measurement from the true (known) value. For these calibration measurements, the mean temperature level is measured using a thermocouple. The reported error is the deviation of the Raman based mean temperature measurement from the thermocouple measurements. A full system calibration is required for each new configuration of the Raman diagnostic system.

The Raman-based calibration rms temperature estimate is ± 1.6% at 296 K and ± 3.1% at 850 K. The Raman temperature measurements reported here illustrate the best-case measurement accuracy that can be expected using the Raman thermometry diagnostic. The calibration plot error bars in Fig. 2 clearly illustrate that the measured rms increases linearly with temperature, as stated above. A linear fit to the rms temperature as a function of the known temperature yields a relation for the expected systematic error in the Raman-based temperature diagnostic which is used to correct the systematic error in the estimated rms in the temperature measurements as described in Locke et al. (2017).

### Raman system density demonstration

**Fig. 3:**
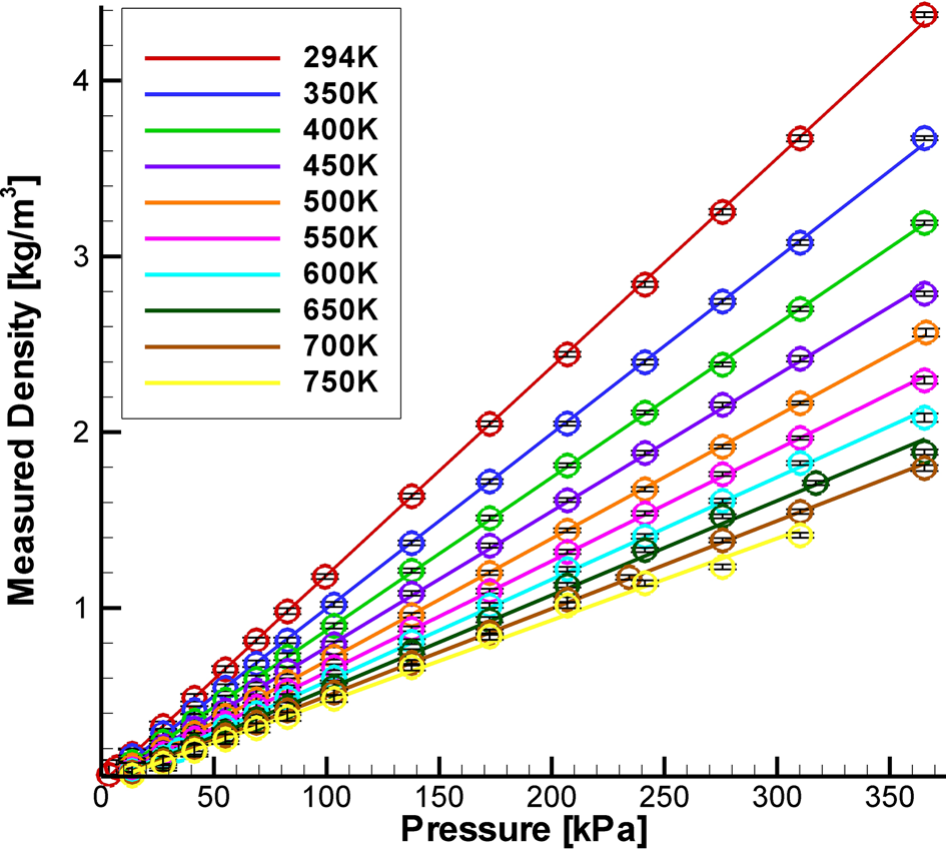
Raman measured gas densities plotted against the set point pressure with the rms density plotted as error bars at each point

A laboratory-scale demonstration of the density measurement capability of the Raman diagnostic was obtained using a cross-shaped stainless steel pressure vessel equipped with four window ports. Two of the opposing window ports were parallel, while the second pair of opposing window ports were tilted at a 45° angle. The Agilite laser beam passed through the tilted pair of window ports in the chamber to avoid back reflections. The collection lenses were aligned on either side of perpendicular set of window ports to collect the Raman scattered light. The vessel was wrapped with electrical heating tape and insulated to reduce heat loss through the metal surfaces. A thermocouple, inserted near the center of the vessel, provided gas temperature measurements essentially at the Raman system probe volume location. The pressure in the vessel was measured using a digital pressure gauge. Raman spectra were collected across a range of pressures and temperatures. For each survey, the temperature in the vessel was held constant while the pressure in the chamber was brought up from a vacuum to approximately 365.4 kPa (53 psia). The Raman spectra were integrated and normalized by the laser pulse energy in order to estimate the gas density. The measured densities are plotted against the pressure in the vessel in Fig. 3. The range of temperatures covered was 294–750 K and the pressure in the vessel covered the range of 1.378 kPa (0.2 psia) to 365.4 kPa (53 psia). The scatter plot illustrates the linear relationship between the gas density in the vessel and the normalized Raman signal energy. The rms variation in the densities are plotted as error bars on the plot. The relative rms variation in gas density is nominally between 1 and 2% over most of the range, increasing to 3–7% below 50 kPa. The error in the estimated density, *σ*_ρ_, relative to the known conditions in the vessel are listed in Table 1. The lowest relative error is at the highest pressure and lowest temperature. The highest relative error is at the lowest pressure and highest temperature condition, where the deviation of the measured density from the known density is not particularly large, however; the absolute density at this condition is very low, yielding the high relative error. The gas in the pressure vessel is assumed to be at isotropic conditions, hence, these plots yield an indication of the error in the density estimates across the full range of conditions measured. The lowest density expected in the jet core measurements in the SHJAR is about 0.5 kg/m^3^, corresponding to approximately 50 kPa in Fig. 3. Hence, the nominal relative error in the density should be < 4% over the expected range of test conditions in the SHJAR.

**Table 1:** Relative error in the density estimates

	σ_ρ_/ρ 300 K (%)	σ_ρ_/ρ 750 K (%)
13.789 kPa (2 psi)	3	28
101.352 kPa (14.7 psi)	0.5	4
310.264 kPa (45 psi)	0.2	2

The vitiation of the air via the hydrogen combustor consumes some of the O_2_ out of the air and produces H_2_O. The area under the Raman spectra is only dependent on the O_2_ and N_2_ present in the vitiated air, since there is no signal detected from the H_2_O over the detected wavelength range. Hence, the density measured by the Raman system in the jet plume is lower than the true gas density since the presence of the H_2_O is not measured.

Density calibrations were performed each run day in the SHJAR facility using probe measurements of the ambient air density and the computed gas density at the nozzle exit. The conditions at the nozzle exit were computed using the plenum gas temperature and pressure along with the temperature compensated ratio of specific heats, γ. The composition of the gas due to the combustion of the hydrogen fuel was also used in the computation of γ and the estimated gas density at the nozzle exit, ${\rho }_{Ideal}$_._ The lower area under the Raman spectra, due to the consumption of the O_2_ during combustion, is equated to the computed vitiated air gas density. Hence, the calibration procedure used for extracting the density from the area under the Raman spectra compensates for the inability of the Raman system to detect H_2_O in the vitiated air. As the vitiated air mixes with the ambient air, the concentration of O_2_ recovers back to the ambient air levels.

### Raman system installation in SHJAR

**Fig. 4:**
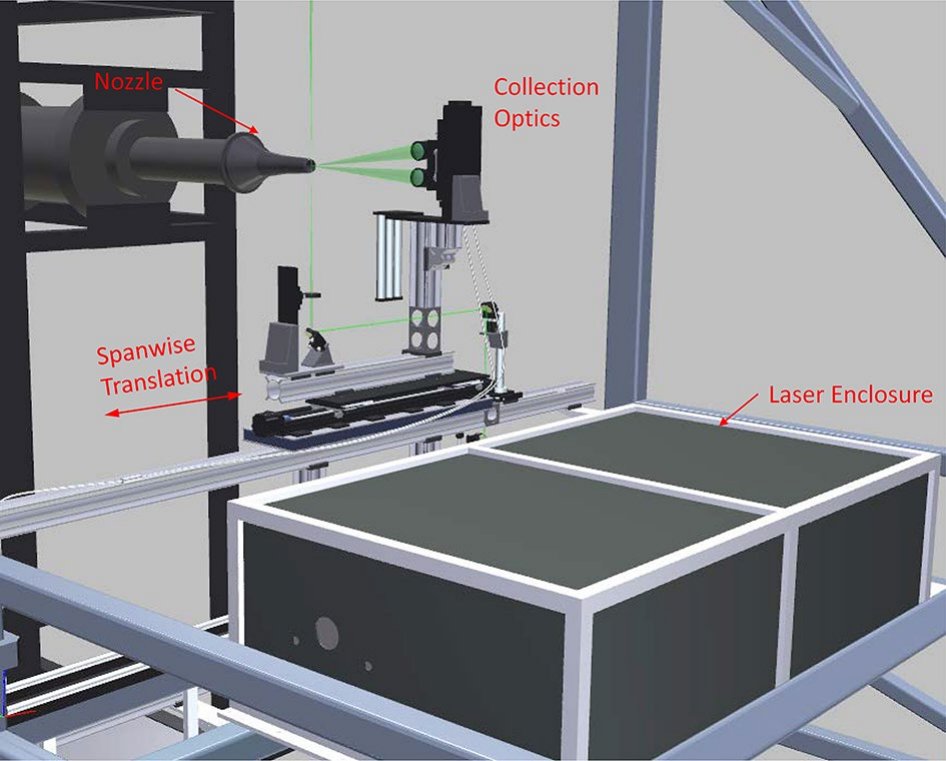
SHJAR and large traverse loaded with the long-pulse laser and Raman collection optics. The Agilite laser is located within the black enclosure

The optical excitation and detection system used to acquire the Raman temperature calibration in the laboratory was also used to acquire the nozzle test data in the AAPL. The long-pulse, Agilite laser, beam insertion optics, detection optics and spectrometer/camera detection systems were transported, installed, and aligned in the AAPL as shown in Fig. 4. Due to its large size, the Agilite laser head had to be mounted inside the frame of the large traverse system. Additionally, the laser head was placed inside of a protective enclosure constructed of metal framing and insulated Plexiglas panels, which is depicted in Fig. 4. The beam exiting from the laser was directed towards the front face of the large traverse where it was turned vertically using a mirror. A catching mirror then turned the laser beam horizontal and parallel to the front face of the large traverse. A final turning mirror directed the laser beam vertical so that it passed vertically through the jet flow field. A 500 mm focal length spherical lens then focused the beam at the center of the flow, downstream of the nozzle’s exit plane. The final turning mirror, lens and the camera collection lenses were mounted on a Velmex translation stage, which provided the transverse surveys of the jet plume. The vertical position of the measurement volume was fixed at the vertical centerline of the jet plume, at the waist of the focused laser beam.

## PIV measurement system

**Fig. 5:**
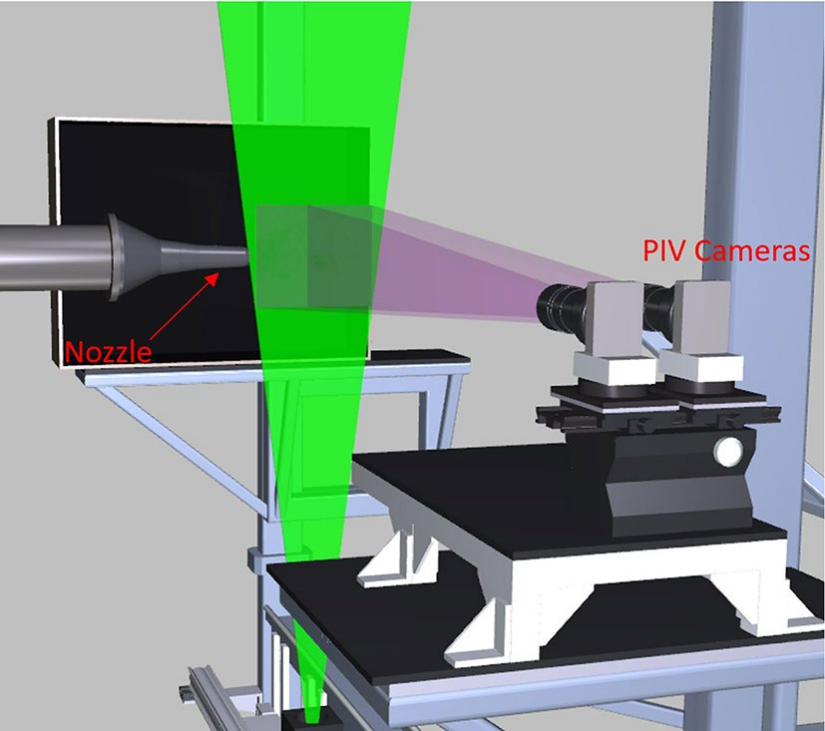
PIV installation in the SHJAR facility showing dual side-by-side cameras along with their overlapping fields-of-view and the laser light sheet

A 2-component PIV system was used to measure the streamwise velocity field down the centerline of the nozzle. In order to maximize the field-of-view while maintaining high spatial resolution PIV vector maps, a dual side-by-side camera configuration was used, as shown in Fig. 5. Both cameras were connected to a single computer system via a CameraLink PCI card and the 400 frame pair data sequences were acquired and streamed to disk at a rate of 2 frame pairs/camera/sec. The pair of 4008 × 2672 pixel ES-11000 cameras were equipped with 135 mm focal length lenses and 8 mm extension tubes and positioned so that their fields of view overlapped by 25.4 mm. The cameras were mounted in portrait mode (4008-pixel axis oriented vertically) yielding two 200 × 300 mm (WxH) imaged fields-of-view, which when combined yielded a 355 × 300 mm field-of-view. The PIV measurement plane was illuminated using a dual head 400 mJ/pulse Nd:YAG laser system with the laser beams formed into vertically diverging 1 mm by 300 mm wide light sheets using cylindrical and spherical lenses. Inter-frame times on the order of 1 microsecond were used to acquire the PIV image pairs. A flat, dot-grid calibration target was used to calibrate and register the two cameras using a fiducial mark in the overlapping region of each cameras’ field of view. The physical registration of the two cameras was used in the setup of the vector processing grids in the left and right camera images so that no interpolation was required in the merging of the left/right vector maps. The PIV system was used to map the jet centerline flow field by traversing in four, 300 mm increments, providing overlapping processed vector maps that were merged into a single 300 × 1220 mm processed vector map.

### PIV vector processing

Sequences of 400 velocity vector maps were acquired at each measurement station. Velocity vector maps for each camera were computed from the image pairs using NASA Glenn in-house PIVPROC software (Wernet 2003). The software utilizes conventional multi-pass PIV cross-correlation processing algorithms and incorporates error detection based on image correlation signal-to-noise ratio. First-pass interrogation region sizes of 64 × 64 pixels on 32 pixel centers and final-pass interrogation region sizes of 32 × 32 pixels on 16 pixel centers were used to process the PIV images. The image sequences were ensemble averaged to provide first- and second-order statistics over the entire measurement plane. Chauvenet’s criterion was used to eliminate any outliers in the ensemble averaging process (Taylor 1982). The final PIV velocity vector maps had a spatial resolution of 1.3 mm and a full-scale measurement error of 1%.

### Flow seeding

For any type of PIV measurements, the fluid motion being measured is marked by the use of small particles. These particles must be sufficiently small so they will have minimal or no slip relative to the fluid (so that their motion is the same as the fluid motion). In addition, all of the fluid must be laden with particles at a concentration high enough that sufficient particles (5–10) are found in an interrogation region of the recorded PIV images. In tests using the SHJAR, two fluid streams are being mixed: the heated core nozzle stream and the ambient air. It is also crucial that the seed be fully mixed and dispersed in the flow upstream of the measurement region in order to ensure good-quality PIV images. Finally, the seed must not be affected by the high temperatures of the gas.

The hot nozzle flow described above was seeded with a refractory seed material, and the ambient air was seeded using a commercial smoke generator. The refractory seed material used for the heated jet flow was 0.4 μm diameter alumina powder. A dispersion of the alumina seed material in 100% ethanol was prepared using a pH stabilization technique (Wernet and Hadley 2016). The alumina/ethanol dispersion was introduced into the flow well upstream of the nozzle using an air-assisted atomizing nozzle. The pH stabilization technique provides highly dispersed, unagglomerated seed particles in the flow. The ambient fluid was seeded with 0.3-μm mineral oil droplets (ρ_*f*_ = 0.84 g/cm^3^) produced by a commercial ‘smoke’ generator. A pair of 1-m-diameter room circulation fans were used to disperse the concentrated smoke emitted by the smoke generator, providing a low velocity (1 m/s), uniformly seeded ambient air around the research jet.

The flow following fidelity of the particles is important in all PIV studies and especially in a supersonic flow investigation. The relaxation time of the alumina particles was computed to be 1.96 μs using the process outlined in Melling (1997). Similarly, the relaxation time for the oil droplets used to seed the ambient flow was determined to be 0.1 μs, which is significantly less than the alumina particles. Assuming Stokes drag law for a sphere, a numerical integration was then performed to compute the alumina particle relaxation distance to a step change in velocity across a shock. The distance for the alumina particles to reach 87% of the flow velocity was computed, yielding a relaxation distance of 2 mm. The PIV subregions used to process the data were on the order of 1 mm. Hence, the alumina particle relaxation in the jet core is masked by the spatial averaging caused by the subregions. The particle relaxation occurs over 1–2 subregions, which results in minimal smearing of the flow features for the data shown here.

## Real-time background-oriented schlieren

**Fig. 6:**
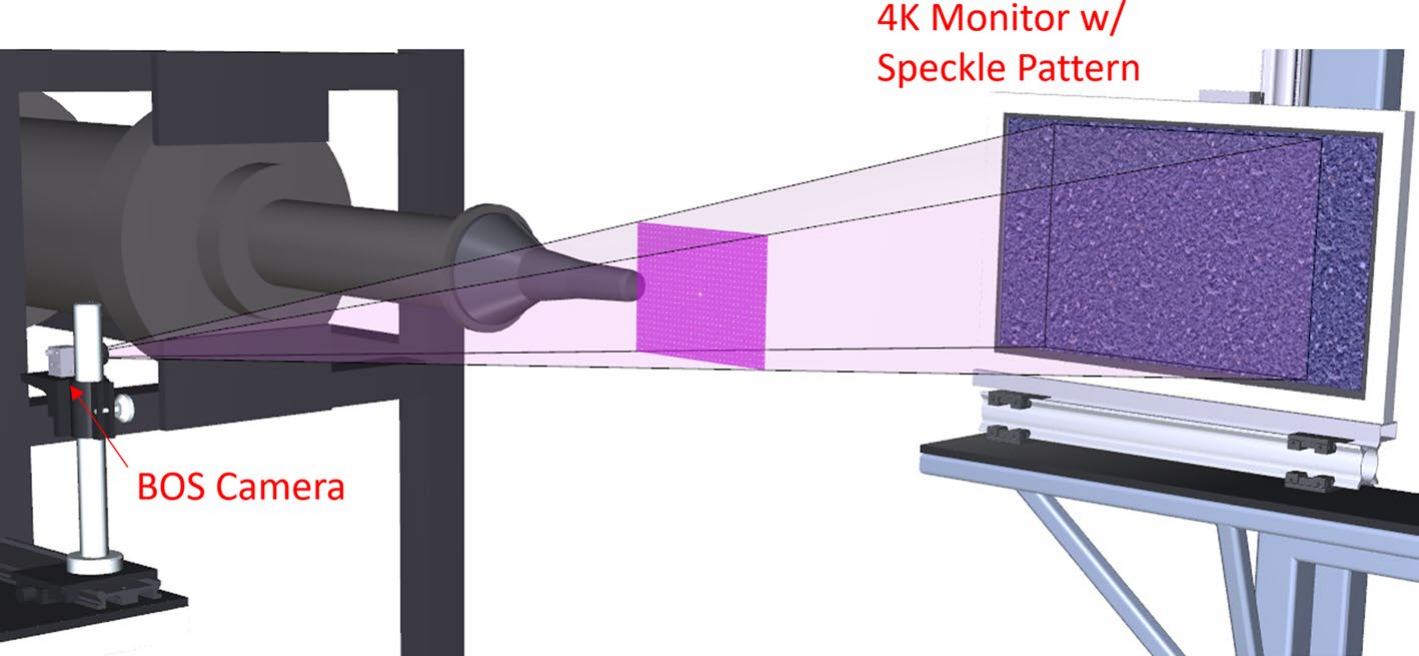
Background-Oriented Schlieren (BOS) system installation in the SHJAR. The field of view of the BOS system at the centerline of the jet is indicated by the magenta rectangle

Background-Oriented Schlieren (BOS) is a widely used technique for measuring density gradients in fluid flows of interest. The attraction of BOS is the simplicity in the setup and data reduction. In this work, two innovations to the BOS technique were employed. First, a GPU based computer was used to acquire and process the BOS image data in real-time, providing a live display of the density gradients in the flow (Wernet 2019a; Wernet 2019b). Secondly, an innovative approach for generating the background speckle patterns was employed. The speckle patterns required for the BOS measurement were displayed on a high definition 4 K resolution computer monitor. Use of the 4 K monitor to display the speckle patterns has three distinct advantages: (1) the speckle patterns can be generated on the computer and displayed directly on the monitor without having to physically construct the speckle pattern; (2) the scale of the speckle pattern can be readily changed to optimize the BOS system performance; and (3) the speckle pattern is self-illuminating, which greatly simplifies implementing the technique in confined environments, or where illumination of a static speckle pattern may be difficult or problematic. A 2.4 × 2 K pixel, GigE interface camera was used to image the speckle pattern displayed on a 1092 mm (43″) diagonal 4 K resolution monitor. The camera was mounted on a shelf on the large traverse system and the monitor was mounted directly across from the camera on a separate shelf, as shown in Fig. 6. The selected speckle pattern contained a continuum of grayscale speckles, not a binary pattern. The processed density gradient maps were processed and displayed at the maximum 12 Hz frame rate of the 5MP GigE camera.

## Baseline CFD analysis

The focus of this paper is on the experimental measurements that were made in order to evaluate and improve CFD and not an extensive CFD investigation. Hence, in order to establish the current state-of-practice for such an experimental configuration, a set of CFD runs were obtained using standard Reynolds-averaged Navier–Stokes (RANS) modeling. CFD simulations of the experimental configuration were run using the Wind-US CFD code (Yoder 2016), a general purpose CFD solver used extensively at NASA GRC for inlet, nozzle, and other propulsion flow simulations. The simulations were run in RANS mode, using the Menter k-ω shear stress transport (SST) model (Menter 1992, 1994). The turbulent Prandtl number, Pr_t_, was set to 0.7 for these cases. The ratio of specific heats was set to 1.4 for all cases considered herein. Within the RANS approximation, the geometry was axially symmetric, which significantly reduced the computational resources required to obtain solutions. Most significantly, the axisymmetric calculations required only 2 dimensions for the calculations—axial and radial. A structured computational grid having 146,455 points across three zones was constructed using the Pointwise software (Pointwise 2013). In the jet plume zone, the grid had 401 axial points by 253 radial points. Grids were packed to solid surfaces such that the reference y + corresponding to the first point off the wall did not exceed 1 along any surface using fully expanded nozzle conditions. The grid was also concentrated axially and radially in the initial part of the jet plume region to resolve the jet mixing and weak expansions and compressions in the jet potential core. Grid sequencing was used whereby a calculation having half the points in each computational direction was compared with a solution using all the points, and indicated virtually no difference between the two solutions. This verifies that the grid was sufficient for the RANS approximation used here. The full grid was used for all the computations discussed in this paper. The nozzle was modeled from slightly upstream of the junction with the facility supply pipe to allow a boundary layer to grow inside the nozzle. The wall surface was set to an adiabatic no-slip surface. In the jet plume zone, the computational grid extended 40 diameters axially and 20 diameters radially from the nozzle exit. A freestream Mach number of approximately 0.02 was set to prevent any convergence difficulties with compressible flow solvers. Previous simulations of jets exhausting into quiescent ambient (Georgiadis et al. 2006) indicate that a freestream flow of this speed would yield jet decay very similar to quiescent air.

## Results and discussion

The set of operating points for which comprehensive data was collected using all of the measurement techniques is shown in Table 2. The set point numbers in Table 2 correspond to the nozzle that was used in the testing; i.e., 140* corresponds to the Mach 1.36 (perfectly expanded) nozzle denoted as SMC015, 166* corresponds to the Mach 1.63 nozzle denoted as SMC017. In addition to the Mach number of the air supply if perfectly expanded, the set point designation number defines the difference between the jet static temperature at the jet exit and the ambient static temperature, Δ*T*. All of the values in Table 2 represent the ideal planned set point conditions and were computed using the gas constant R for clean air and* γ* = 1.4. The actual set point operating conditions for each type of data being collected (PIV, BOS, Raman) were different due to: variations in the water content in the combusted air; day-to-day variations in the ambient temperature; and the fact that the data were collected on different days. The ambient temperatures varied from 268 to 281 K over the duration of the test campaign. One final note, there are inconsistencies in the Set Point naming since additional set points were added to the matrix while the test was underway.

**Table 2:** Operating set points with operating conditions

Nozzle	Set Point	Mach	ΔT [K]	^T^s,Ideal ^[K]^	^T^T,Plenum ^[K]^	^P^T,Plenum ^[kPa]^	p_Ideal_[kg/m^3^]	U_j_ [m/s]
SMC017	1661	1.63	0	285	442	441.24	1.1981	544
SMC017	1665	1.63	133	408	657	441.24	0.8065	669
SMC017	1662	1.63	233	508	799	441.24	0.6626	745
SMC017	1664	1.36	233	514	715	298.75	0.6626	580
SMC015	1401	1.36	0	278	396	298.75	1.1981	457
SMC015	1405	1.36	133	418	578	298.75	0.8196	549
SMC015	1402	1.36	233	516	715	298.75	0.6626	605
SMC015	1404	1.63	233	510	799	441.24	0.6626	721
SMC015	1403	1.36	333	618	852	298.75	0.5561	662

### Background-oriented schlieren results

**Fig. 7:**
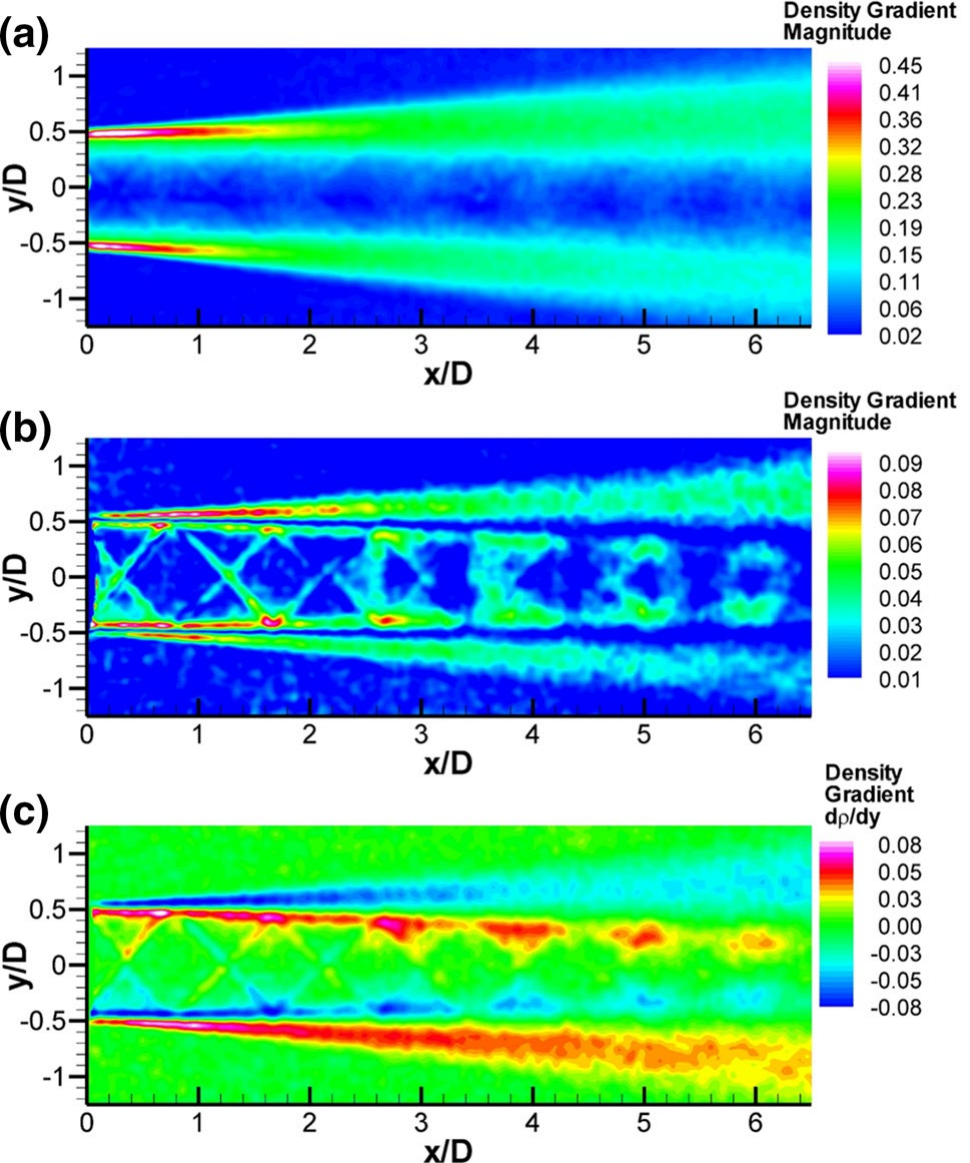
BOS density gradient maps for SMC015: **a** Density gradient magnitude at Set Point 1402, **b** density gradient magnitude at Set Point 1401, and **c** y-component of the density gradient at Set Point 1401

The SMC nozzles were designed using perfectly expanded conditions, yet when installed in the SHJAR, they still yielded weak shocks. The real-time BOS system, with the live display, provided a means for adjusting the rig operating pressure and jet exit temperature to yield a nearly shock-free potential core flow. Small adjustments (up to approximately 10 kPa (~ 1%) for the Mach 1.63 case) to the rig set point pressure were required to achieve a pseudo shock-free exit. BOS measurements of the jet plume flow were acquired before Raman or PIV measurements were collected. The qualitative BOS measurements were used to determine the locations of the Raman measurement points across the shear layer. Because the number of Raman temperature measurement locations was time-limited, these BOS images were very helpful in setting and optimizing these locations. Examples of the BOS results are shown in Fig. 7 for SMC015 at both Set Point 1402 (ΔT = 233 K) and Set Point 1401(Δ*T* = 0 K) as color contour maps of density gradient magnitude. The density gradient magnitude is used since it incorporates both the* x*- and* y*-components of the measured density gradients and best illustrates the series of weak compressions and expansions in the potential core. For the 1402 case (Δ*T* = 233 K) shown in Fig. 7a, the highest gradients in density are across the shear layer near the nozzle exit. Across the potential core, the density gradients are relatively flat as denoted by the blue region. The density gradient map also shows that the matched pressure at the nozzle exit still results in a series of weak compressions and expansions. These weak compressions and expansions are enclosed within the top and bottom shear layers denoted by the bright red regions in the density gradient maps. For the Set Point 1401 result in Fig. 7b, the processed BOS data again reveals a series of weak compressions and expansions in the jet plume. The scale range of the density gradient fluctuations at Set Point 1401 is much smaller than the one used in Fig. 7a for Set Point 1402, due to the small-scale fluctuations when the temperature gradient between the jet core and the ambient is set to 0. An interesting feature is observed at Set Point 1401, and in all of the Δ*T* = 0 cases, there is a region of minimal density gradient magnitude in the middle of the shear layer, as denoted by the dark blue regions running down the center of the shear layer. Actually, the density gradient magnitude plot is masking the sign of the density gradient across the shear layer. Figure 7c shows only the* y*-component of the density gradient map for Set Point 1401, which illustrates the change in sign in the density gradient across the shear layer. The blue region running down the center of the shear layer in the density gradient magnitude plot in Fig. 7b actually delineates the region where the density gradient changes sign, and therefore defines the minima in density across the shear layer. The shear layer is defined as the region where the high-speed jet flow mixes with the quiescent ambient air. For Set Point 1401, where the temperature of the jet core flow is matched to the ambient, the mixing of the high-speed jet flow with the quiescent ambient air actually results in a measurable increase in the local gas temperature. The heat of mixing from the shear builds up faster than the thermal diffusion rate of the gas, resulting in the localized increase in the gas temperature along the entire length of the shear layer, which results in the observed low-density trough in the BOS data.

### General Raman and PIV results

**Fig. 8:**
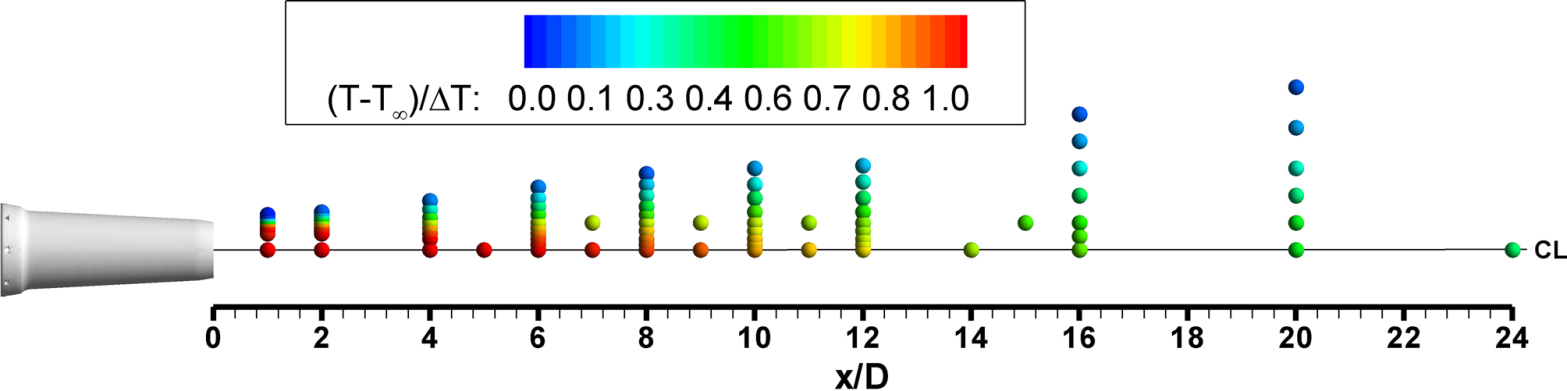
Nominal measurement grid and an example of Raman measured temperatures for SMC015 Set Point 1402

Temperature data were acquired over a wide range of flow temperatures and Mach numbers as listed in Table 2. In this paper, results for three of these conditions are presented: 1) SMC015 at Set Point 1402 (M1.36, Δ*T* = 233 K); 2) SMC015 at Set Point 1401 (M1.36 Δ*T *= 0 K); and 3) SMC017 at Set Point 1664 (M1.36, Δ*T *= 233 K). Figure 8 illustrates a typical Raman temperature measurement grid, which is actually a combination of three separate profiles: centerline (r/D = 0), lip-line (r/D = 0.5) and radial profiles at 9 axial stations. A thin line is drawn depicting the nozzle centerline. The symbols in the scatter plot are colored by their non-dimensional temperature. The symbols used in Fig. 8 are too large to clearly resolve all of the radial measurement points. The radial profiles overlapped both the centerline and lip-line profiles. An ambient temperature point was acquired prior to the start of each run day using the Raman measurement system. These ambient temperatures and their rms temperature variations provide a check on the performance of the Raman measurements in the jet flow facility.

The BOS measurements provided a clear picture of the extent of the jet shear layer and facilitated the optimization of the Raman radial profile measurement grids. The measurement grids for each Set Point are in general not identical, depending on the growth of the shear layer and length of the potential core. Raman centerline measurements were acquired at 15 axial locations (x/D = 1, 2, 4, 5, 6, 7, 8, 9, 10, 11, 12, 14, 16, 20 and 24) for all of the temperature surveys. The lip-line profiles were acquired at 12 axial stations (x/D = 1, 2, 4, 6, 7, 8, 9, 10, 11, 12, 15, and 20). Radial profiles were acquired at axial stations of x/D = 1, 2, 4, 6, 8, 10, 12, 16 and 20. The spacing of measurement points was adjusted to cover the jet shear layer. Typically, 10 points were acquired in each radial profile. After the unexpected drop in the density gradient was observed in the BOS data, additional points were added to the Δ*T* = 0 Set Point radial surveys.

When collecting the Raman scattered light, an edge filter is used to block the Rayleigh scattered light, which occurs at the laser line wavelength of 532 nm. Some Rayleigh light may still leak through the filter, depending on the intensity of the light. Typically, there are no particles in the flow when acquiring the Raman data. When particles and/or condensation are present in the air, they scatter light at the laser line wavelength. The particle scattered light is orders of magnitude brighter than the Rayleigh light scattered by molecules. Hence, particles in the flow can be problematic for processing the Raman spectra. In cases where there is some particle contamination in the flow, additional checks on the Raman spectra signal amplitude, especially around the laser line wavelength, are used to remove any particle-laden spectra from the ensemble of measurements used to compute the mean and rms values. In this experiment, even the Δ*T* = 0 case resulted in some condensation in the jet core, yet Raman temperature measurements were still possible under these conditions. More aggressive filtering was applied to ensure that no particle contaminated spectra were being used to compute the mean and rms quantities; however, the extra filtering had no impact on the mean and rms temperature estimates from the Raman spectra, confirming that the Raman temperature estimates were unaffected by the particle scattered light. The density estimates are directly dependent on the scattered light signal level. When processing the Raman spectra to estimate density, more aggressive filtering was required, yielding only 700 spectra in the ensembles for estimating the density. This was done as a precaution against biasing the density estimates. Reducing the number of good spectra below 700 had no noticeable impact on the computed density estimates.

The streamwise PIV dual-camera system had a field of view of 355 × 300 mm and was traversed in four 300 mm increments to map the centerline plane of the nozzle jet plume. For the low temperature cases, some condensation was observed in the jet plume. The condensation actually occurs in the shear layer where the moist ambient air mixes with the cold jet potential core air. The condensation reaches a maximum at the end of the potential core where the shear layers converge. Although present, the condensation did not adversely impact the PIV measurements.

### SMC015 set point 1402 PIV and Raman results

**Fig. 9:**
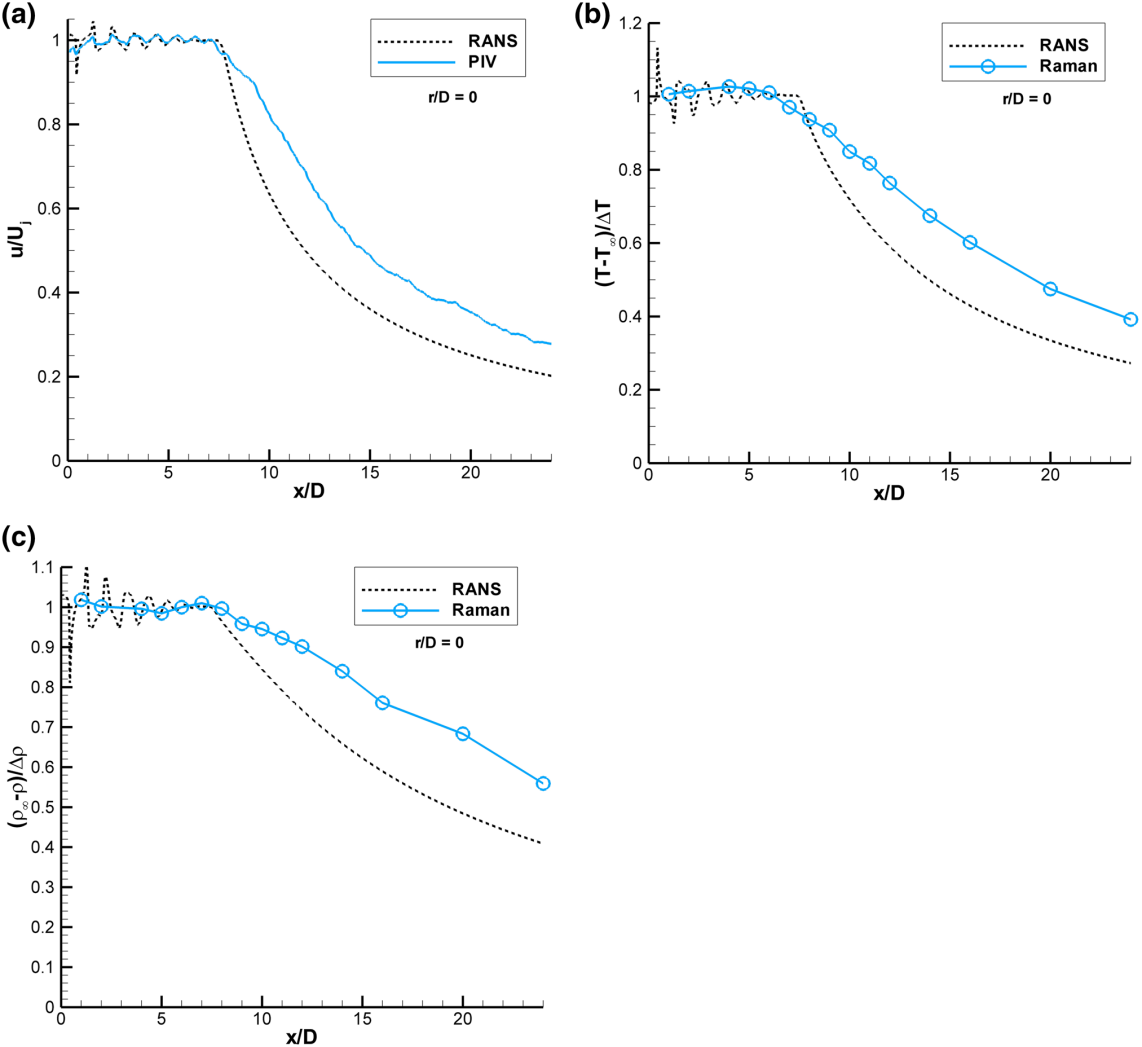
Comparison of the normalized PIV and Raman centerline measurements of: **a** velocity; **b** temperature; **c** density (solid lines) versus RANS (dashed lines) for SMC015 at Set Point 1402

Figure 9 shows a comparison of the PIV and Raman measured centerline (r/D = 0) flow properties against a RANS solution for SMC015 at Set Point 1402 (Δ*T *= 233 K, M = 1.36). Figure 9a shows the centerline normalized velocity data where the PIV and RANS data agree very well along the centerline within the potential core of the jet, matching both the length, shape and location of the weak shock structures. The RANS solution has an abrupt drop at the end of the potential core and predicts a faster decay of the velocity beyond the potential core (x/D > 8) which is a known issue for RANS models in computing compressible jet flows. The Raman data yield both the mean and rms fluctuations in the gas temperature and density. The Raman temperature data are non-dimensionalized using the computed static temperature at the jet exit and the ambient temperature (T-T_∞_)/(T_s Ideal_-T_∞_). Figure 9b compares the non-dimensionalized Raman temperature measurements with the RANS solution. The low-resolution measurement grid of the Raman centerline profile does not capture the weak shock structures along the jet centerline that are present in the RANS solution. The RANS solution again shows an abrupt drop in temperature at the end of the potential core, as was observed in the velocity comparison. The processed density estimates are non-dimensionalized using the ideally expanded density at the nozzle exit and the ambient gas density (ρ_∞_-ρ)/(ρ_∞_-ρ_Ideal_). The centerline density data are plotted against the RANS solution in Fig. 9c, where the density exhibits the same general trends observed in the temperature comparison.

**Fig. 10:**
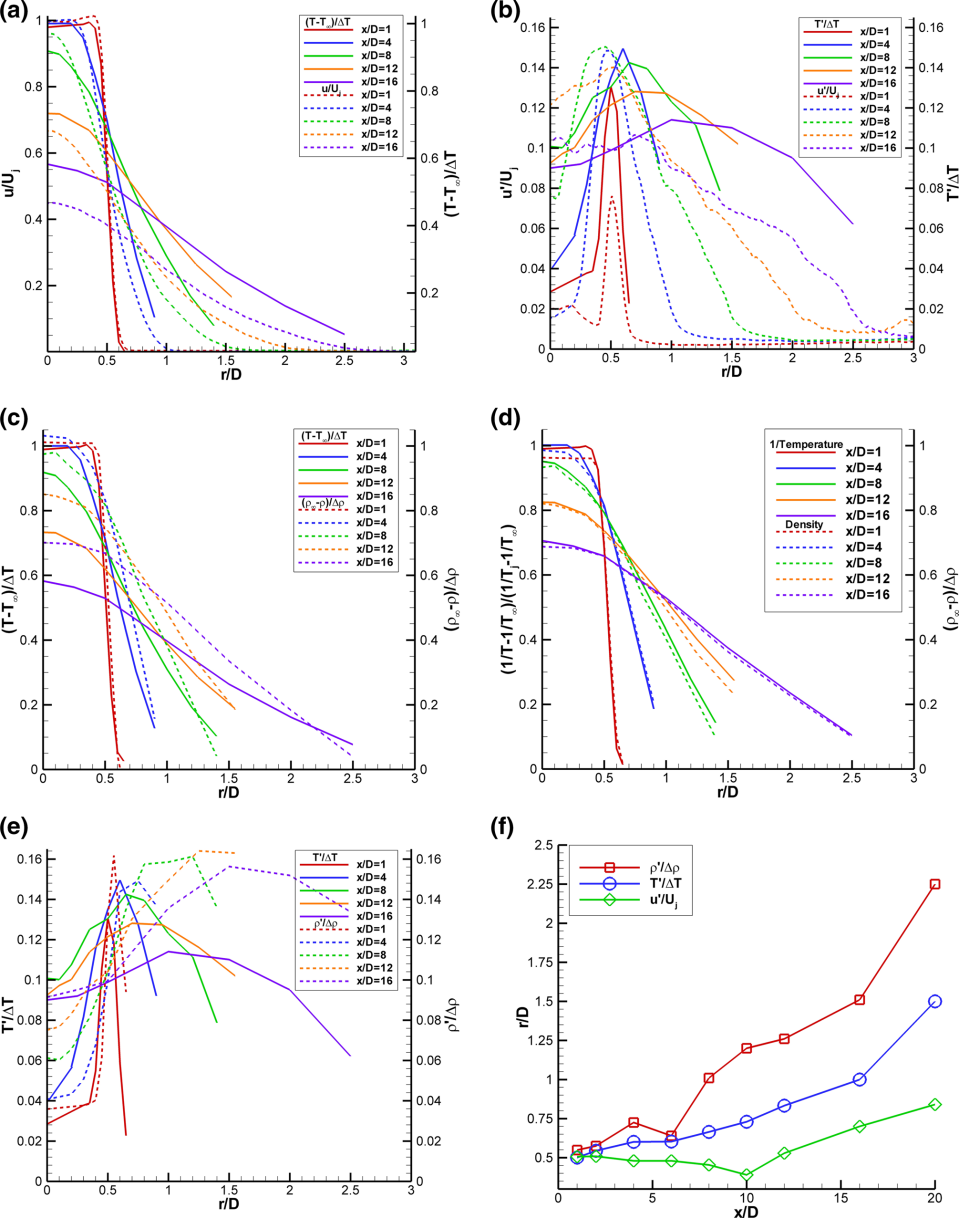
SMC015 at Set Point 1402 **a** Comparison of radial profiles of u/U_j_ to (T-T_∞_)/ΔT;** b** comparison of radial profiles of u′/U_j_ to T′/ΔT;** c** Comparison of radial profiles of (T-T_∞_)/ΔT to (ρ_∞_-ρ)/Δρ;** d** Comparison of radial profiles of normalized inverse temperature (1/T-1/T_∞_)/(1/T_j_-1/T_∞_) to (ρ_∞_-ρ)/Δρ;** e** Comparison of radial profiles of T’/ΔT to ρ’/Δρ; f) Radial locations of the peak rms intensities for u, T and ρ at each axial station

Figure 10a shows the radial PIV normalized velocity data (dashed lines) plotted along with the non-dimensional Raman temperature (solid lines) data for Set Point 1402. The radial temperature and velocity profiles are similar for x/D < 4. Beginning at x/D = 4, the non-dimensional temperature profiles spread more slowly than velocity. The trend becomes more pronounced with increasing axial distance. Figure 10b shows radial profiles of the normalized rms velocity data (dashed lines) plotted along with the normalized rms temperature (solid lines) data. Close to the nozzle exit, the u′/U_j_ values are likely underestimated due to the spatial averaging of the PIV correlation subregions. The normalized rms velocities generally peak symmetrically about the lip-line; however, the normalized rms temperatures peak at progressively larger radial locations with increasing axial distance. The peak in T′/ΔT at larger radial locations with increasing distance downstream is corroborated by the growth in the thermal mixing layer thickness observed in Fig. 10a.

Figure 10c shows radial profile comparisons of the Raman non-dimensional temperature (solid lines) and density (dashed lines) at increasing axial stations along the jet plume. For the supersonic jet flow, the pressure outside of the potential core is essentially equal to the ambient pressure; hence, the temperature and density measurements should be redundant and exhibit similar behavior. However, the non-dimensional density profiles exhibit different decay shapes across the shear layer compared to the non-dimensional temperature profiles, which is due to the inverse relationship of density on gas temperature. The two profiles decay at different rates, eventually merging back together outside of the shear layer where the gas temperature has reached the ambient temperature and is no longer changing. Similar differences in the non-dimensional temperature and density profiles were observed in the RANS solution.

A better comparison of the temperature and density profiles in the constant pressure region of the shear layer is obtained by plotting the radial profiles of normalized inverse temperature (1/T-1/T_∞_)/(1/T_j_-1/T_∞_) (solid lines) against the non-dimensionalized density (dashed lines), as shown in Fig. 10d. Close to the nozzle exit x/D < 4, the profiles are nearly identical. The difference between the two curves for x/D ≤ 4 inside of the potential core (r/D < 0.5) is probably due to pressure fluctuations. Otherwise, the non-dimensionalized inverse temperature profiles agree very well with the normalized density profiles, illustrating that the measurements in such regions of the flow where the pressure is equal to the ambient value are in fact redundant. The small deviations between the profiles are well within the stated error bands of the measurements.

The rms temperature and density data in Fig. 10e are each non-dimensionalized by the difference between the value at the jet exit and the ambient value. The magnitude of the two non-dimensional rms quantities are nominally the same. However, the non-dimensional rms temperatures peak closer to the lip-line of the nozzle, while the non-dimensional rms densities peak at increasingly larger radial distances with increasing axial location. Figure 10f shows the r/D locations of the peak in the normalized rms u′/U_j_, T′/ΔT and ρ′/Δρ at each axial station. The trajectories of the rms peaks show the velocity fluctuations remain close to the lip line while the peak rms temperature and densities occur at increasingly larger radial locations. The results shown here agree with the previous work of Panda et al. (2004), where Rayleigh scattering measurements showed that the density shear layer ρ′/Δρ was outboard of the velocity shear layer u′/U_j_ in heated jets.

**Fig. 11:**
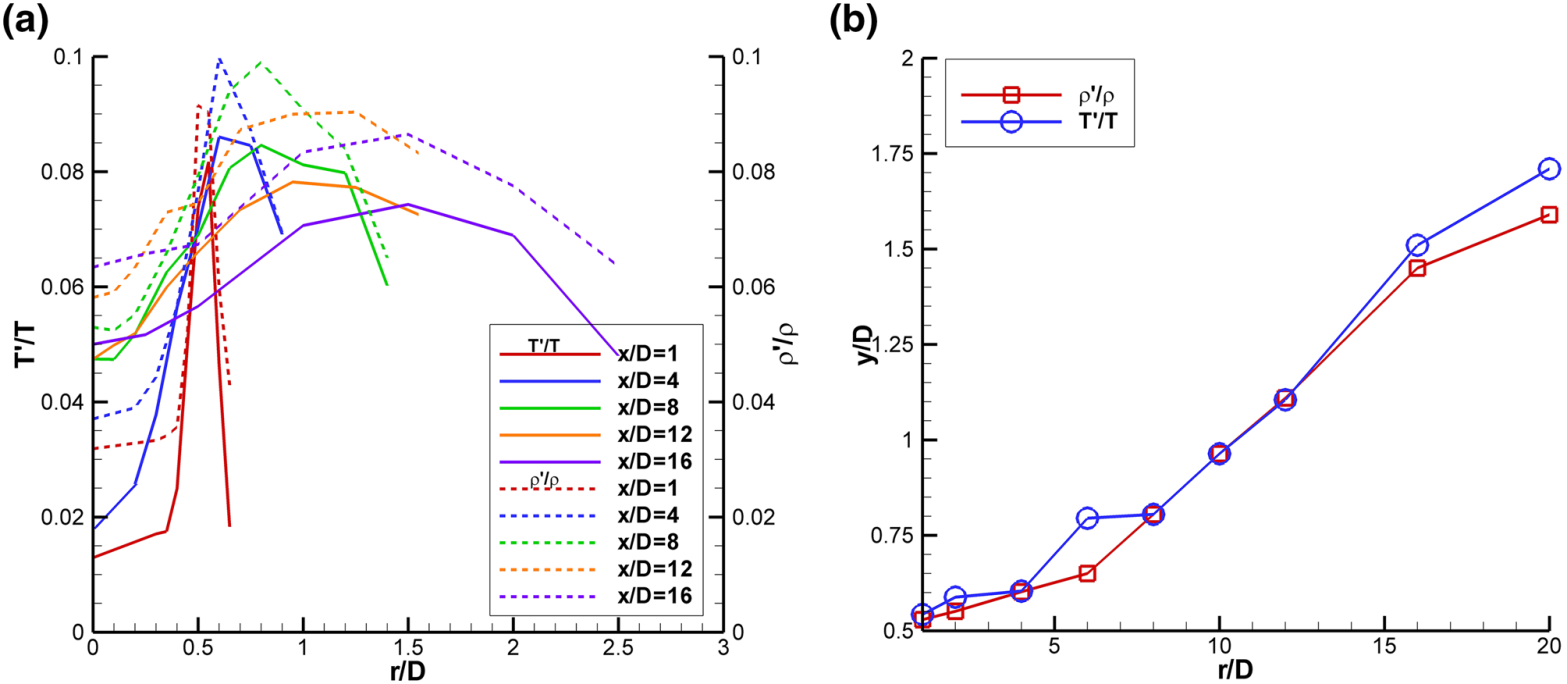
SMC015 at Set Point 1402 **a** Radial profiles of rms temperature intensity and rms density intensity; **b** Radial locations of the peak rms intensity in temperature and density as a function of axial station

The rms data in Figs. 10b, e are normalized by the constant reference values as stated above. The local intensities of the turbulent fluctuations of each of the quantities can be obtained by normalizing the rms values by local mean values of each of the variables. Figure 11a shows radial profiles of the rms temperature intensity (T′/T) and rms density intensity (ρ′/ρ), which both peak at the same radial locations. Hence, the expected coupling of the density and temperature fluctuations is confirmed, since the static pressure does not vary significantly across the shear layer. Figure 11b shows the radial locations of the peak rms intensities in temperature and density as a function of axial station. The locations of the peak intensity values for both temperature and density are nearly identical for all axial stations. The rms density intensities exhibit higher peak values than the rms temperature intensities due to the nominally larger error in the density estimates compared to the temperature estimates.

### SMC015 set point 1401 PIV and Raman results

In many jet studies, the nozzle exit temperature is either significantly above or below the ambient air temperature. For the two different nozzle geometries tested here, a ΔT = 0 set point was used for each nozzle, meaning that the jet core static temperature was matched to the ambient air static temperature. Examination of the PIV radial velocity profiles revealed nothing unusual occurring in the shear layer. However, as stated previously, the BOS data exhibited a localized increase in the gas temperature in the jet shear layer for both nozzles at the ΔT = 0 set point. Hence, the resolution and spacing of the Raman temperature radial profile measurement grids for the Δ*T *= 0 cases were altered to further investigate the feature observed in the BOS data. Conventional approaches for non-dimensionalizing the temperature data, such as that used thus far in this report, become problematic when Δ*T* = 0. Hence, a different approach for non-dimensionalizing the data was required.

**Fig. 12:**
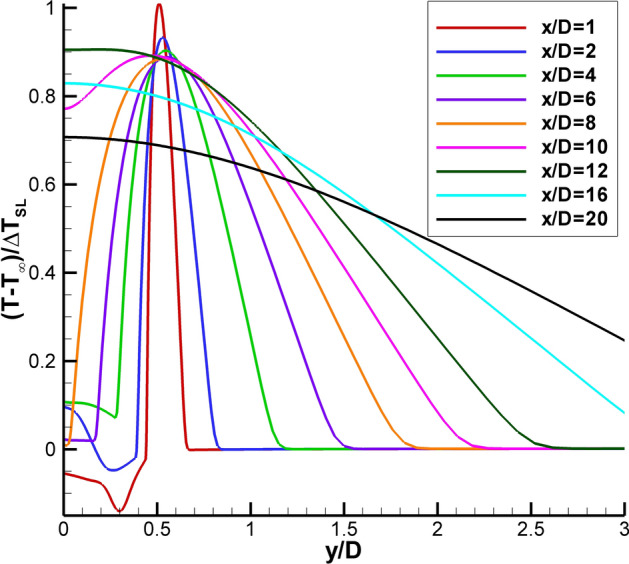
Non-dimensional radial profiles of the RANS solution for SMC015 at Set Point 1401

An alternative approach for presenting the Δ*T* = 0 case data was developed by examining the temperature rise across the shear layer in the RANS solution for SMC015 at Set Point 1401, where M = 1.36. Since the standard Δ*T *(based on the difference between the nozzle exit temperature and the ambient temperature) cannot be used, the authors propose that the appropriate characteristic temperature difference is the peak temperature across the shear layer at x/D = 1 (T_SL_ = 316.6 K), relative to the ambient temperature (T_∞_ = 288.8 K). Applying this shear-layer-based characteristic temperature difference, Δ*T*_SL_, the non-dimensional radial RANS profiles are plotted in Fig. 12, where the desired non-dimensional peak temperature of 1 is obtained at the center of the shear layer and the profiles tend to 0 in the ambient. There is some undershoot in the jet core region (x/D < 4), which can be ignored for the purposes of examining the phenomenon of interest across the shear layer.

**Fig. 13:**
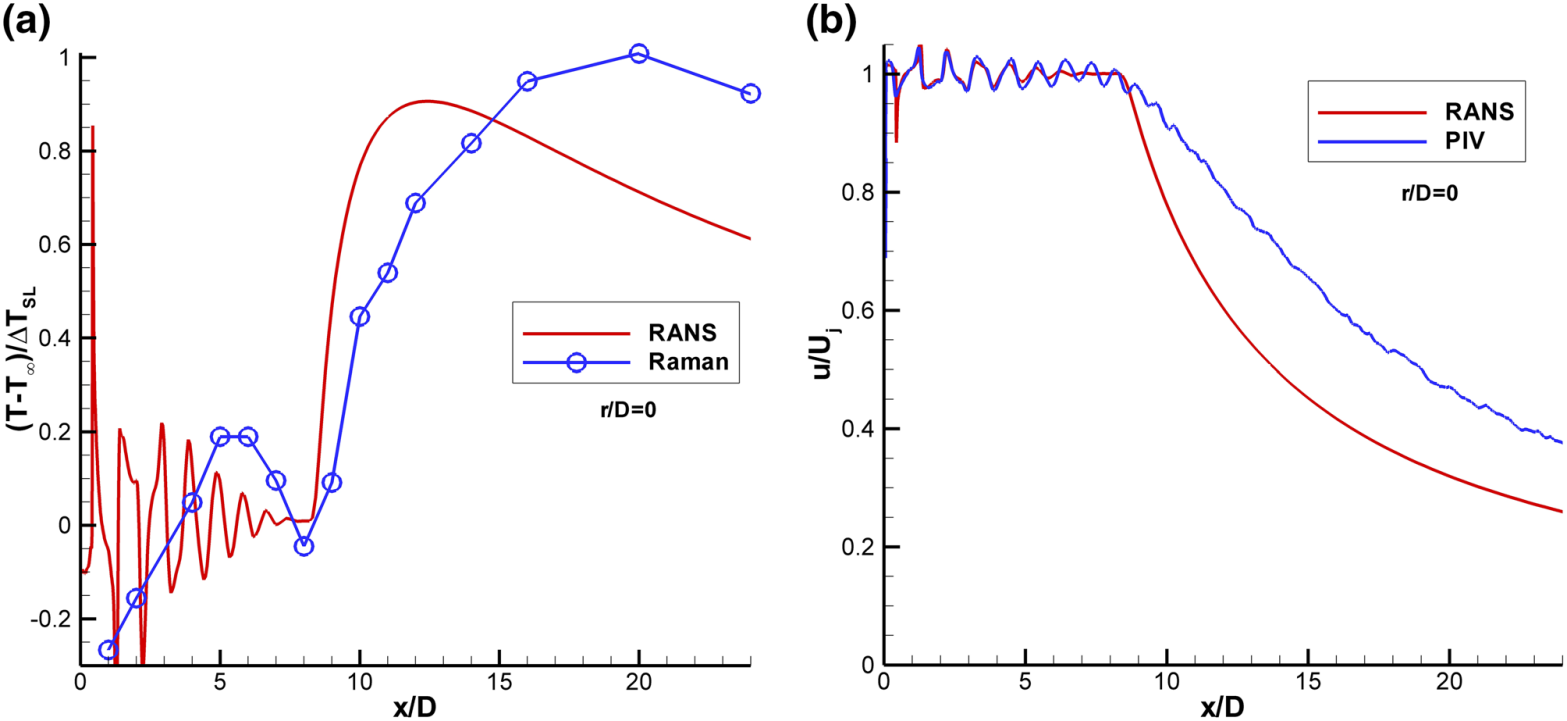
Comparison of RANS against Raman for SMC015 at Set Point 1401: **a** non-dimensional centerline temperature profiles; **b** normalized velocity profiles

Applying the same ΔT_SL_-based normalization approach to the Raman temperature profiles enables comparison with the RANS result for the centerline profile as shown in Fig. 13a. For the Set Point 1401 Raman data set, the peak temperature in the shear layer, T_SL_, was determined to be 301.4 K when T_amb_ = 277.1 K. The centerline profile shows an increase in temperature up to approximately 5 D followed by a drop in temperature before the final rise in temperature at the end of the potential core. Initially, this spike in temperature was believed to be the result of condensation in the nozzle plume. However, after comparison with the RANS solution it appears that the undersampling of the nozzle plume via the coarse Raman temperature measurement grid in the potential core yields an aliased depiction of the true sinusoidal temperature profile, due to the weak shocks present in the potential core. The RANS predicts a slightly shorter potential core length than that measured by Raman. The faster decay of the RANS velocities was also observed when compared to PIV, as shown in Fig. 13b. The PIV data also indicates that the weak shock oscillations persist past the end of the mean potential core, which has been reported in other supersonic jet flow studies (Wishart and Krothepalli 1994; Gross et al. 2010; Feng and McGuirk 2016). At the end of the time-averaged potential core, the shear layers have merged and the high turbulence in this region should dominate the flow. However, examination of the individual velocity vector maps reveals that the length of the potential core changes, due to instabilities in the jet plume. At times when the potential core is elongated, the shock cells persist further downstream (the number of shock cells increases) and when the potential core is shorter, the shock cell train is shorter (decreasing the total number of shock cells). Averaging the ensemble of measurements yields a mean flow field with weak oscillations past the end of the potential core observed here. A more thorough discussion of these phenomena are discussed in Wernet et al. (2020).

**Fig. 14:**
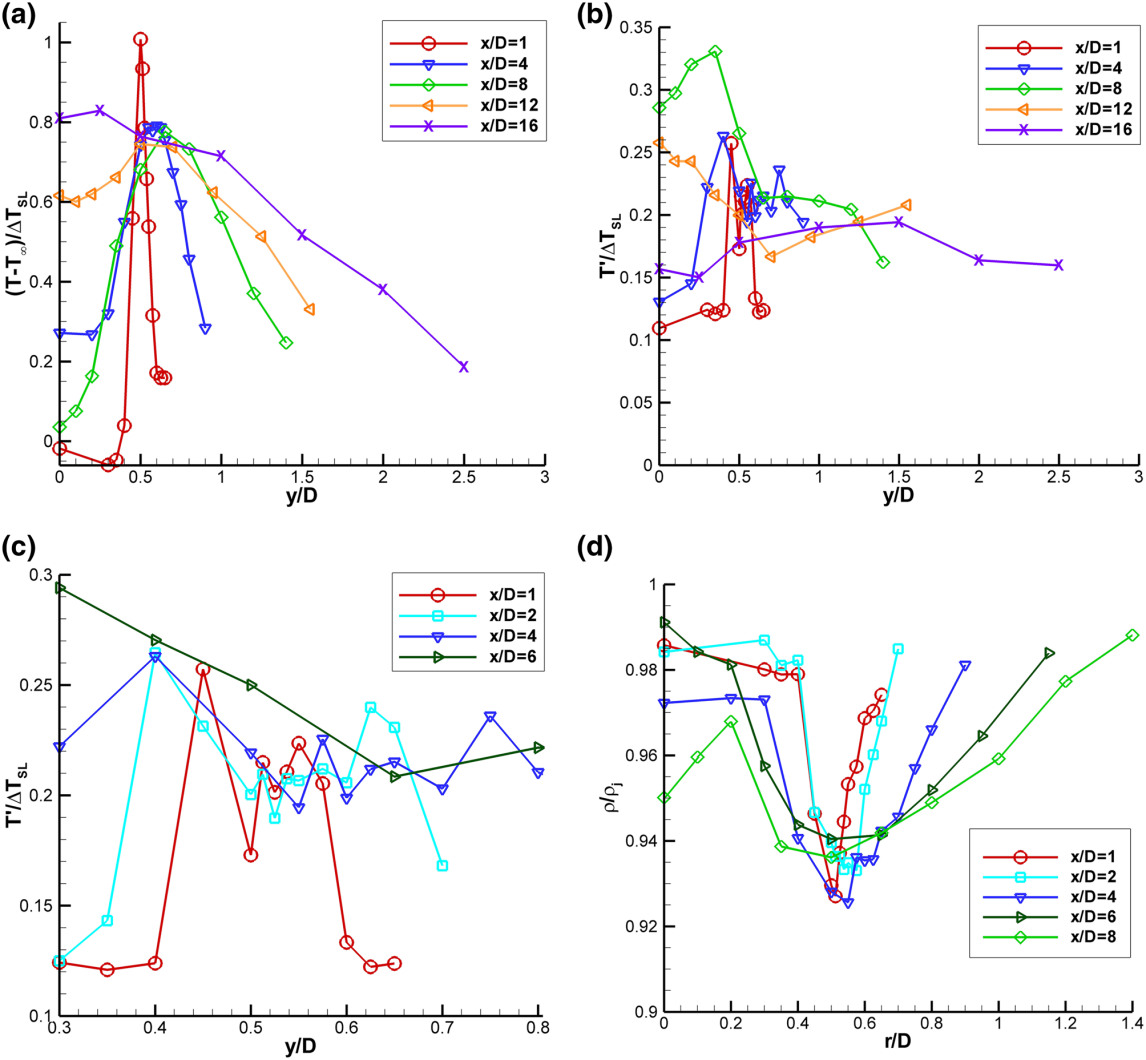
Non-dimensional Raman data for SMC015 at Set Point 1401: **a** radial profiles of mean temperature; **b** radial profiles of rms temperature; **c** expanded region of the rms temperature radial profiles centered about the lip line; and **d** normalized mean density measurements across the shear layer

Radial profiles of the mean and rms Raman temperature data normalized by ΔT_SL_ for SMC015 at Set Point 1401 are shown in Fig. 14. The radial profiles have a high concentration of points surrounding the expected peak temperature in the shear layer as a function of x/D. The temperature does spike across the shear layer, with the sharpest peaks near the jet exit, as shown in Fig. 14a. The rms profiles in Fig. 14b show that there is actually a drop in the rms temperature when the temperature peaks in the shear layer. The minima are best observed in Fig. 14c, where an expanded region of the first 4 axial station radial profiles centered about the nozzle lip-line are shown. The drop in rms temperature at the peak temperature in the shear layer suggests that this is the source location of the gas heating, hence the gas is of more uniform temperature at the source, since it has not yet mixed with gas in the jet core or the ambient. The gas is heating from the turbulent mixing and resultant dissipation along the center of the shear layer, the region of peak shear. The slow diffusion of the heat results in the observed localized temperature increase. Figure 14d shows the mean density normalized by the density at the nozzle exit plotted across the shear layer for the first 5 axial stations. The normalized density profiles exhibit a drop in density at the center of the shear layer, which coincides with the peak temperatures measured across the shear layer in Fig. 14a. The Raman measured trough in density along the shear layer is the cause of the drop in density gradient magnitude observed in the BOS measurements at Set Point 1401.

### SMC017 set point 1664 PIV and Raman results

Almost all of the centerline Raman temperature profiles were acquired at relatively low resolution inside the potential core since measuring the temperatures within the weak shock pattern at the perfectly matched jet exit conditions were not of high importance. For SMC017 at Set Point 1664, the nozzle was run off-design, at an over-expanded condition, with a nozzle pressure ratio corresponding to that of a Mach 1.36 perfectly expanded case and Δ*T* = 233 K. The nozzle supply total pressure would reach equilibrium for a nozzle exit to throat area ratio corresponding to the Mach 1.36 nozzle, and hence the effective diameter of the nozzle is given by:

1 $$D_{eff} = D\sqrt {\frac{{\left( {A/A^{*} } \right)_{M = 1.36} }}{{\left( {A/A^{*} } \right)_{M = 1.63} }}}$$

which yields D_eff_ = 47 mm.

**Fig. 15:**
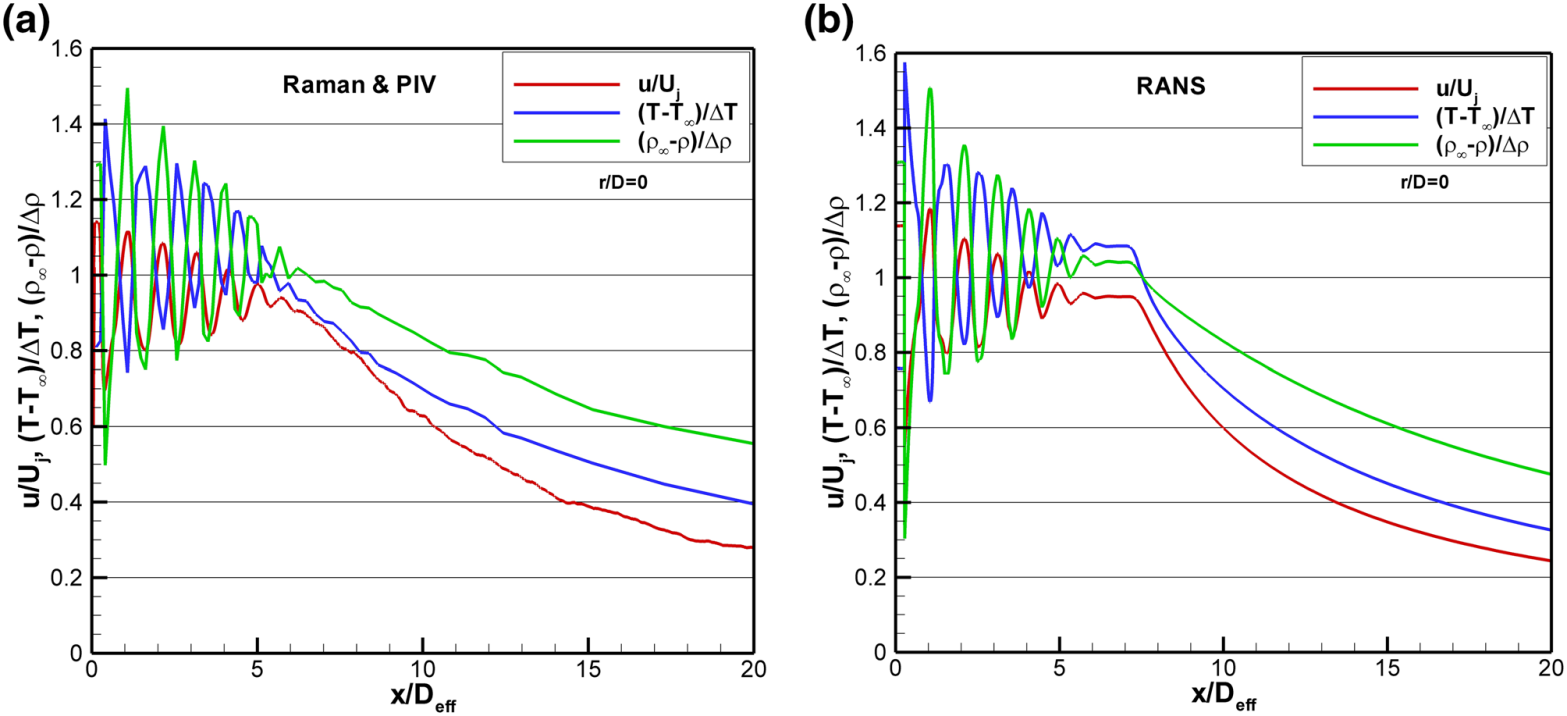
Comparison of non-dimensional mean velocity temperature, and density for the over-expanded jet condition on SMC017 at Set Point 1664 for **a** Raman and PIV; **b** RANS

A high-resolution Raman centerline measurement grid was used to profile the jet centerline at Set Point 1664. The measurement grid used progressively larger spacings, beginning with 6.35 mm increments out to 254 mm, then switching to 12.7 mm increments out to 406.4 mm followed by 25.4 mm increments. The processed, non-dimensional temperature and density data are plotted along with the streamwise PIV normalized velocity data in Fig. 15a, where D_eff_ is used to normalize the axial coordinate. Examination of the centerline velocity, temperature and density all indicate that the potential core length is approximately 6 D_eff_. Because of the significantly over-expanded conditions in the set point 1664 case, not only are there oblique shock waves and expansion waves, but the first compression results in a normal shock near the nozzle exit and at the centerline. From the RANS solution, this normal shock, (also referred to as a Mach disk) results in a loss of total pressure such that the downstream total pressure is 0.881 times the plenum total pressure. Further, while the perfectly expanded Mach number for the set point 1664 conditions is 1.36, with the drop in total pressure caused by the normal shock, the new equilibrium Mach number is 1.267. The equilibrium values of jet properties, namely velocity, temperature, and density, are 0.948, 1.036, and 0.965 times the perfectly expanded jet potential core values, respectively. In other words, these are the mean values that the post-normal shock centerline jet quantities oscillate upon through the rest of the potential core. Note that the reference values used in the non-dimensionalizations for both experimental and computational results in Fig. 15 employ the perfectly expanded jet quantities. The sinusoidal oscillations of the non-dimensional velocity and temperature within the potential core are exactly out of phase, as expected. The non-dimensional density peaks are also out of phase with the non-dimensional temperature peaks due to their inverse temperature dependence. The magnitude of the non-dimensional density peaks are the largest, followed by the oscillations in the non-dimensional temperature profile. The non-dimensional velocity peaks are the lowest, possibly due to the spatial averaging of the PIV data. Downstream of the potential core, the temperature and density decay a little more slowly than the velocity, as also observed in the Set Point 1402 case in Fig. 9. For comparison, a RANS solution for Set Point 1664 is shown in Fig. 15b. The RANS solution shows slightly higher oscillations in temperature and density and also slightly over-predicts the length of the potential core, out to x/D_eff_ = 8. The decay of the flow properties downstream of the potential core agrees very well between experiment and the RANS prediction. Both the flow measurements of velocity, temperature and density and the RANS prediction denote the existence of the double shock peak in the 2^nd^ shock cell as previously reported by Raman (1998). The double shock peak is most readily visible as a “shoulder” on the second temperature peak (≈1.5 D_eff_), but is also evident in the minima in the velocity profiles around 1.5 D_eff_.

**Fig. 16:**
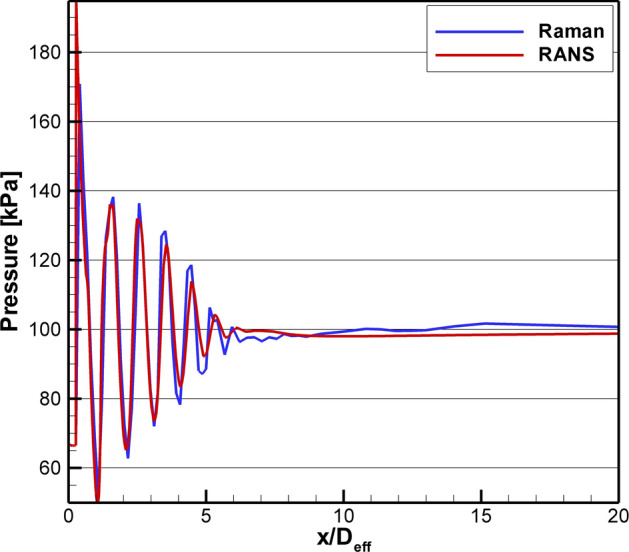
Comparison of centerline pressure computed from the Raman temperature and density data to the pressure from the RANS solution for SMC017 at Set Point 1664

Inside the potential core of the over-expanded jet flow, the significant difference in the magnitude of the temperature and density oscillations across shock cells measured via Raman indicates that they are not redundant. In this region of the flow, the true value of the simultaneous measurement of temperature and density is realized, where the local pressure can be computed. Figure 16 shows the computed pressure from the data in Fig. 15a plotted along with the pressure from the RANS solution. The agreement between the experiment and the predictions is quite good. The pressure oscillates within the potential core and then equilibrates to the ambient pressure for x/D_eff_ > 6.5. Probe measurements within the potential core of supersonic jets are incapable of accurately extracting both the local pitot pressure and the local static pressure at precisely the same spatial location. Hence, these measurements represent the first ever high spatial-resolution, non-intrusive measurements of local gas pressure in the potential core of an over-expanded jet.

## Conclusions

Two 50.8-mm-exit-diameter supersonic nozzles with perfectly expanded Mach numbers of 1.36 and 1.63 were tested across a range of temperatures. The temperature difference between the jet core and the ambient were held at fixed values of 0, 133 K, 233 K, 333 K. PIV, BOS, and Raman spectroscopic measurements of both mean and rms temperature and density were acquired in the jet flows. The acquired data set significantly expanded the spatial extent and properties of the flow field that were measured compared to previous studies in supersonic jets, adding off-centerline characterization of the flow on the nozzle lip-line and across the shear layer and measurements of static temperature and density.

The RT-BOS system enabled the optimization of the SHJAR operating pressure and temperature settings to yield a nearly shock-free, perfectly-matched flow condition. The RT-BOS data quickly revealed an unanticipated feature in the ΔT = 0 case, a low-density region running down the center of the shear layer. The phenomena was observed in both nozzles operating at the perfectly matched condition and the jet exit temperature matched to the ambient gas temperature. This low-density region is coincident with the Raman measured high-temperature region, which is due to the local mixing and heating of the gas at a faster rate than the thermal energy can diffuse. The small magnitude of the flow feature is not normally observed in flows where the nozzle exit temperature is significantly higher or lower than the ambient air. BOS data facilitated the optimization of the Raman radial measurement profiles to capture the same phenomena in the shear layers for the temperature matched cases. The Raman technique enabled both verifying the existence of the low-density regions and measurement of the increased temperatures confirmed that the heating was due to the shear between the high-speed core flow and the ambient air.

Rotationally Resolved Raman spectroscopy provided unobtrusive measurements of both mean and rms temperature and also mean and rms density across the jet plume. The Raman temperature and density along with PIV measurements were compared against RANS solutions of the nozzle flows, generally showing good agreement at the lower Mach number nozzle and increasing under prediction of the potential core length with the higher Mach number nozzle. The centerline PIV measurements generally showed a relative decrease of the potential core length with increasing jet exit temperature and increasing Mach number. The PIV data also showed the existence of weak shock oscillations past the end of the potential core. An explanation for these persistent oscillations was proposed.

The Raman data confirmed the BOS measurements of the temperature spike along the centerline of the shear layer for the ΔT = 0 cases. The RANS solution also illustrated this feature across the shear layer. Outside of the potential core the pressure in supersonic jet flows is essentially equal to the ambient pressure; hence, the temperature and density measurements obtained via Raman in these regions are redundant. Plots of non-dimensionalized inverse temperature and density confirmed that the pressure is constant across the shear layer. The rms temperature intensity and rms density intensity were demonstrated to peak at the same radial locations across the shear layer, illustrating the consistency of the two different flow-field property measurements extracted from the Raman spectra. The velocity and temperature profiles did not decay at the same rate across the shear layer.

Combining a high spatial resolution Raman centerline survey with PIV data collected on a Mach 1.36 over-expanded jet yielded well-resolved mappings of the sinusoidal oscillations in temperature, density and velocity along the jet centerline. The velocity and temperature oscillations were exactly out of phase as expected. The variation in the magnitude of the temperature and density oscillations within the potential core suggested that the pressure was not constant. The simultaneous gas temperature and density measurements within the over-expanded jet potential core were used to compute the oscillations in local gas pressure, which to the best of the author’s knowledge, has not been previously reported in the literature. Overall, the magnitude of the measured velocity, temperature and density oscillations and their mix out rates agreed very well with the RANS solutions.

## Acronyms

AAPL: Aeroacoustic Propulsion Lab
BOS: Background Oriented Schlieren
CFD: Computational Fluid Dynamics
GRC: Glenn Research Center
LDV: Laser Doppler Velocimetry
PIV: Particle Image Velocimetry
RANS: Reynolds averaged Navier–Stokes
RMS: Root Mean Square
SHJAR: Small Hot Jet Acoustic Rig
SRS: Spontaneous Raman Scattering

## Symbols

A: Nozzle exit area [mm^2^]
A*: Nozzle throat area [mm^2^]
C_P_: Specific heat of air [kJ/kg K]
D: Nozzle diameter = 50.8 mm
D_eff_: Effective nozzle diameter for the SMC017 nozzle operated at the perfectly match condition for the SMC014 nozzle
M: Mach number
M_ideal_: Ideally expanded jet Mach number
Pr_t_: Turbulent Prandtl number
r: Radial coordinate [mm]
R: Gas constant [J/kg K]
T′: Rms temperature [K]
T_j_: Temperature at jet exit [K]
T_SL_: Peak temperature across the shear layer [K]
T_∞_: Ambient static temperature [K]
u: Axial velocity component [m/s]
u′: Rms in axial velocity component [m/s]
U_j_: Axial velocity component at jet exit [m/s]
v: Vertical component of velocity [m/s]
w: Spanwise component of velocity [m/s]
x: Axial distance from nozzle exit [mm]
y: Vertical coordinate from nozzle centerline [mm]
z: Spanwise distance from nozzle centerline [mm]
Δ*T*: Difference between jet exit temperature and ambient temperature = (T_j_-T_∞_) [K]
Δ*T*_SL_: Difference between peak temperature across the shear layer and ambient temperature = (T_SL_-T_∞_) [K]
Δ*ρ*: Difference between ambient air density and the jet exit density = (ρ_∞_-ρ_Ideal_) [kg/m^3^]
Δ*ρ*_SL_: Difference between ambient air density and the minimum density across the shear layer = (ρ_∞_-ρ_SL_) [kg/m^3^]
γ: Ratio of specific heats
*ρ*: Gas density [kg/m^3^]
*ρ*_f_: Fluid density [g/cm^3^]
*ρ*: Rms in gas density [kg/m^3^]
*ρ*_SL_: Gas density in the minimum across the shear layer [kg/m^3^]
*ρ*_Ideal_: Gas density at the nozzle exit [kg/m^3^]
*ρ*: Ambient gas density [kg/m^3^]
*σ*_*ρ*_: Measurement error in the density estimates [kg/m^3^
